# Identification and analysis of *UGT* genes associated with triterpenoid saponin in soapberry (*Sapindus mukorossi* Gaertn.)

**DOI:** 10.1186/s12870-024-05281-4

**Published:** 2024-06-21

**Authors:** Mingzhu Zhou, Jialin Fan, Yuhan Gao, Chunyuan Zheng, Yuanyuan Xu, Liming Jia, Xinmin An, Zhong Chen

**Affiliations:** 1 State Key Laboratory for Efficient Production of Forest Resources, Key Laboratory of Silviculture and Conservation of the Ministry of Education, Ministry of Education of Engineering Research Centre for Forest and Grassland Carbon Sequestration, College of Forestry, Beijing Forestry University, P. O. Box 407, No.35 Qinghua East Road, Haidian District, Beijing, 100083 China; 2 College of Forestry, Guangxi University, Nanning, 530004 China; 3 National Engineering Research Center of Tree Breeding and Ecological Restoration, College of Biological Sciences and Biotechnology, Beijing Forestry University, Beijing, 100083 China

**Keywords:** *Sapindus mukorossi* Gaertn., Triterpenoid saponins, UGT, Expression pattern

## Abstract

**Background:**

Soapberry (*Sapindus mukorossi*) is an economically important multifunctional tree species. Triterpenoid saponins have many functions in soapberry. However, the types of uridine diphosphate (UDP) glucosyltransferases (UGTs) involved in the synthesis of triterpenoid saponins in soapberry have not been clarified.

**Results:**

In this study, 42 *SmUGT*s were identified in soapberry, which were unevenly distributed on 12 chromosomes and had sequence lengths of 450 bp to 1638 bp, with an average of 1388 bp. The number of amino acids in SmUGTs was 149 to 545, with an average of 462. Most SmUGTs were acidic and hydrophilic unstable proteins, and their secondary structures were mainly α-helices and random coils. All had conserved UDPGT and PSPG-box domains. Phylogenetic analysis divided them into four subclasses, which glycosylated different carbon atoms. Prediction of *cis-*acting elements suggested roles of *SmUGT*s in plant development and responses to environmental stresses. The expression patterns of *SmUGT*s differed according to the developmental stage of fruits, as determined by transcriptomics and RT-qPCR. Co-expression network analysis of *SmUGT*s and related genes/transcription factors in the triterpenoid saponin synthesis pathway was also performed. The results indicated potential roles for many transcription factors, such as *SmERF*s, *SmGATA*s and *SmMYB*s. A correlation analysis showed that 42 *SmUGT*s were crucial in saponin synthesis in soapberry.

**Conclusions:**

Our findings suggest optimal targets for manipulating glycosylation in soapberry triterpenoid saponin biosynthesis; they also provide a theoretical foundation for further evaluation of the functions of *SmUGT*s and analyses of their biosynthetic mechanisms.

**Supplementary Information:**

The online version contains supplementary material available at 10.1186/s12870-024-05281-4.

## Background

Soapberry (*Sapindus mukorossi*) is a deciduous tree of the Sapindaceae family, which is mainly distributed in east, south, and southwest China, and is used as a source of daily chemicals, medicines, and biomass energy. In one study, the triterpenoid saponin content in the peel of soapberry ranged from 4.14% to 27.04% [1, 2]. Triterpenoid saponins are the main soapberry components with medicinal value and play an important role in plant resistance to bacteria. In addition, because of their surfactant properties saponins are used in the manufacture of chemicals such as shampoo [3]. These specialized metabolites are glycoside compounds formed by condensation of a hydrophobic triterpenoid saponin and one or more hydrophilic glycogroups [4]. They are mainly present in plants in families such as Araliaceae, Leguminosae, Cucurbitaceae, and Umbelliferae [5]. Saponins participate in physiological processes such as communication, defense, and perception in plants [6], and have antioxidant, anti-inflammatory, and anticancer effects [7, 8]. They can be used in the production of herbicides, insecticides [9], and emulsifiers [10]. Saponins are often the main bioactive components of several plants. The main pharmacological components of *Panax ginseng*, *Panax quinquefolius*, and *Panax notoginseng* are saponins of the ginsenoside type [11]. Glycyrrhizin in *Glycyrrhiza glabra* is also a pharmacologically relevant saponin [12]. Mogrosides from *Siraitia grosvenorii* are used in the manufacture of natural sweeteners [13]. The structural diversity of triterpenoid saponins has led to widespread interest in their applications in medicine, food, daily chemical products, agriculture, and other fields. The complex structure and frequently low content of saponins in plant biomass make it challenging to directly extract, isolate, or obtain them through chemical synthesis, thus restricting their wide application. Biotechnology methodologies have been used to study the biosynthetic pathways of triterpenoid saponins to increase the content of target metabolites or produce them using synthetic biological methods.

The biosynthesis of triterpenoid saponins begins with isopentenyl pyrophosphate (IPP, C5) and dimethylallyl pyrophosphate (DMAPP, C5). IPP and DMAPP are synthesized through two distinct pathways: the mevalonate (MVA) pathway in the cytoplasm and the 2-C-methyl-d-erythritol-4-phosphate (MEP) pathway in plastids. The MVA pathway utilizes acetyl-CoA as its initial substrate and undergoes a six-step condensation reaction to generate IPP. In contrast, the MEP pathway involves a seven-step reaction to synthesize IPP from pyruvate and glyceraldehyde 3-phosphate as starting substrates. The MVA pathway plays a key role in triterpenoid saponin biosynthesis. Subsequently, IPP and DMAPP are condensed by geranyl pyrophosphate synthase (GPS) to produce geranyl pyrophosphate (GPP, C10). GPP is then catalyzed by farnesyl pyrophosphate synthase (FPS); this is followed by the addition of a second IPP unit to produce farnesyl pyrophosphate (FPP, C15). The condensation of two FPP molecules by squalene synthase (SS) results in the formation of squalene (C30), which is converted to 2,3-oxidosqualene through epoxidation. Finally, 2,3-oxidosqualene undergoes cyclization mediated by oxidized squalene cyclase (OSC), oxidation mediated by cytochrome P450 enzymes, and glycosylation mediated by uridine diphosphate glucosyltransferase (UGT) [5, 14–16].

UGT glycosyltransferases of the glycosyltransferase 1 family (GT1) transfer active glycosylates from uracil nucleoside diphosphate sugars to small-molecule receptors in plants [17], and glycosyltransferases associated with triterpenoid saponin biosynthesis in soapberry belong to this class. The sequence of the N-terminal region of these enzymes is highly diverse and is responsible for the recognition of multiple substrates, whereas the C-terminal region contains a conserved plant secondary product glycosyltransferase (PSPG) motif. The UGT-catalyzed glycosylation of triterpenoids is commonly considered the final stage in the biosynthesis of triterpenoid saponins; it plays a crucial role in generating diversity and biological activity. A typical triterpenoid saponin contains 2 to 5 glycans, which usually form a sugar chain at the C-3 and/or C-28 position of the saponin. Triterpenoid saponins glycosylated at C-4, C-16, C20, C-21, C-22, and/or C-23 are also present. Common glycans include glucose (Glc), galactose (Gal), glucuronic acid (GlcA), rhamnose (Rha), xylose (Xyl), and arabinose (Ara), as well as other five- or six-membered sugars. The number, composition, and position of sugar chains on the triterpenoid skeleton impact the bioactivity, stability, solubility, and cellular uptake of triterpenoid saponins [5].

*UGTs* in plants are organized according to their amino acid sequence identity into gene families (identity > 40%) and subfamilies (identity > 60%) [6]. In all, 107 *UGT*s have been identified in *Arabidopsis thaliana*, distributed in 14 groups (A-N) [18]; 182 *UGT*s in soybean, distributed in 16 groups (A-P) [19]; 147 *UGT*s in maize, distributed in 17 groups (A-Q) [20]; and 241 *UGT*s in apple, distributed in 16 groups (A-P) [21]. The members of different subfamilies can have different functions. For example, most UGT73s have glycosylation activity at C-3 or C-28, whereas UGT74s and UGT94s typically catalyze glycosylation at C-3 [5]. Regarding functions, *UGTPg1* produces ginsenoside F1 through region-specific glycosylation of C20-OH of proginsenodiol (PPD) and proginsenotriol (PPT). *UGTPg100* specifically glycosylates C6-OH of PPT to produce ginsenoside Rh1 [22]. Similarly, *PG3-O-UgT1* from *Panax quinquefolium* catalyzes the conversion of proginsenediol into ginsenoside Rh2 [23, 24]. Several UGT enzymes are related to plant resistance in *Barbarea vulgaris* [25]. A number of studies have focused on the *UGT* genes in *Medicago truncatula* [26], *Centella asiatica* [27], cucumber [28], *Glycyrrhiza glabra* [12], and *Siraitia grosvenorii* [13].

Research on triterpenoid saponins in soapberry has focused on their extraction, separation, and application. For example, Zhang and Jia investigated the pharmacological effects of soapberry [29]. Peng et al*.* optimized the extraction of saponins from soapberry [30]. Ma evaluated the use of soapberry extract as an herbicide [31]. However, there has been little research on triterpenoid saponin biosynthesis and the key enzymes in glycosylation. In this study, we constructed a phylogenetic tree and analyzed the expression patterns of *SmUGT*s related to triterpenoid saponin synthesis, based on available genomic and transcriptomic data of soapberry [32]. The findings provided theoretical support for functional research on soapberry genes related to triterpenoid saponin synthesis.

## Results

### Identification and structural analysis of *SmUGT* genes

**Fig. 1:**
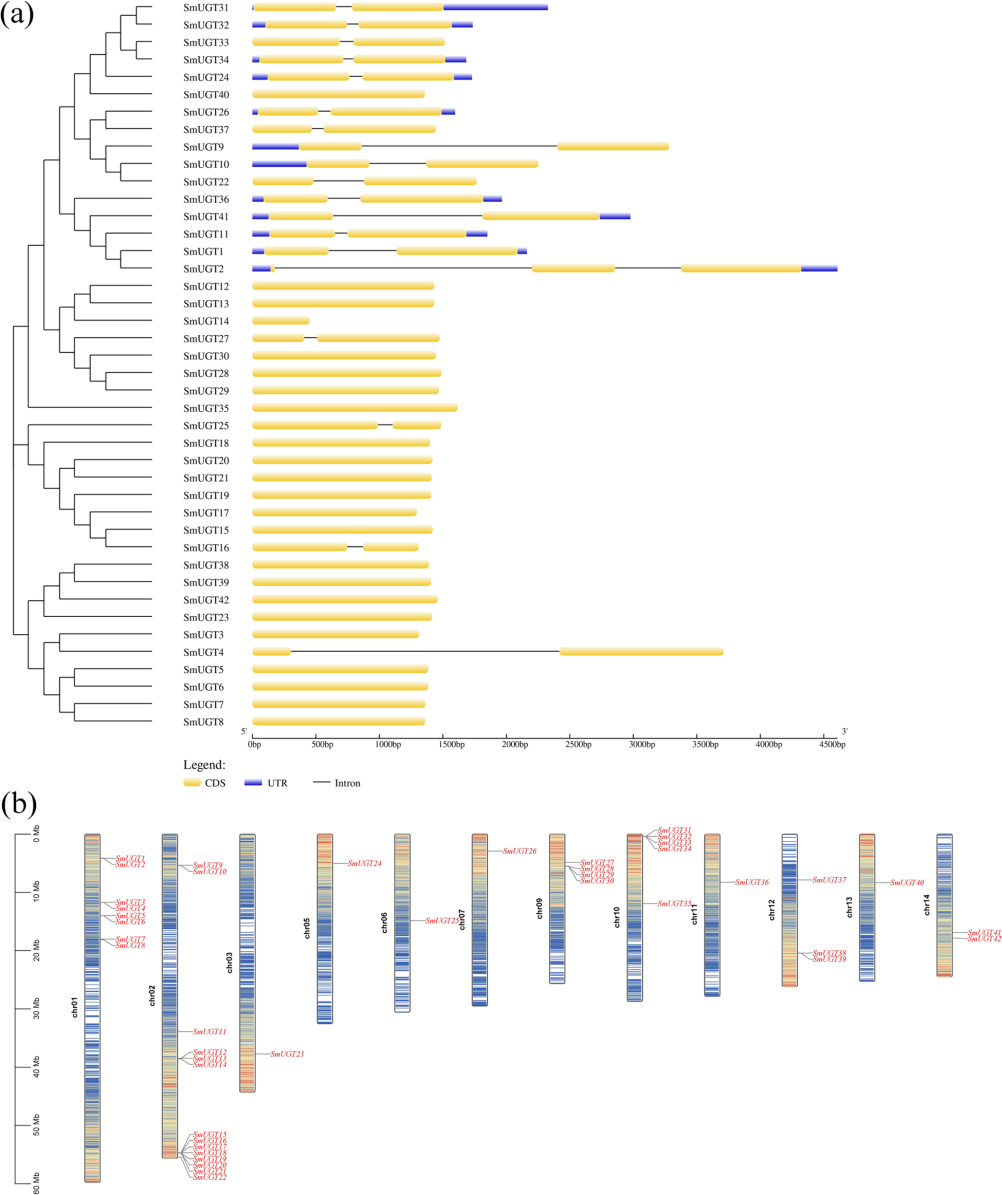
Structures and chromosome locations of *SmUGT* genes. **a** Exon and intron structures of *SmUG*T genes. The CDS is the coding region of the protein; the UTR is the untranslated region, a non-coding fragment at each end of the mRNA molecule; the intron, also known as spacer order, refers to a fragment of a gene or mRNA mole. **b** Chromosomal locations of *SmUGT*s. 42 *SmUGT*s were unevenly distributed on 12 chromosomes

In total, 42 *SmUGT*s were identified, which were successively named *SmUGT1* to *SmUGT42*. Gene structure analysis (Fig. 1) showed that 23 genes did not contain introns. Eighteen genes had one intron and one gene had two introns; most genes containing introns clustered into one group. A protein domain analysis (Figure S1) showed that the SmUGTs contained the Pfam: UDPGT domain typical of UGT proteins, which were close to the C-terminus, indicating structural consistency. The 42 *SmUGT*s were distributed among 12 chromosomes, and chr02 had the most genes (14).

### Physicochemical properties of SmUGT proteins

The average length of these genes was 1388 bp, the longest was 1638 bp, and the shortest was 450 bp (Table 1). Except for *SmUGT14*, the genes were > 1000 bp in length. The number of amino acids ranged from 149 to 545, with an average of 462. Other than SmUGT14 and SmUGT16, the SmUGT proteins were composed of > 400 amino acids. The average molecular weight was 51,764.76 Da and the theoretical isoelectric points of 41 of the proteins were < 7, indicating acidity. That of SmUGT14 was 8.43, indicating alkalinity. In all, 33 were hydrophilic and 9 were hydrophobic; 11 were stable (instability coefficient < 40) and 31 were unstable (instability coefficient > 40). Most were predicted to localize in the cytoplasm, chloroplasts, and nucleus.

**Table 1.**
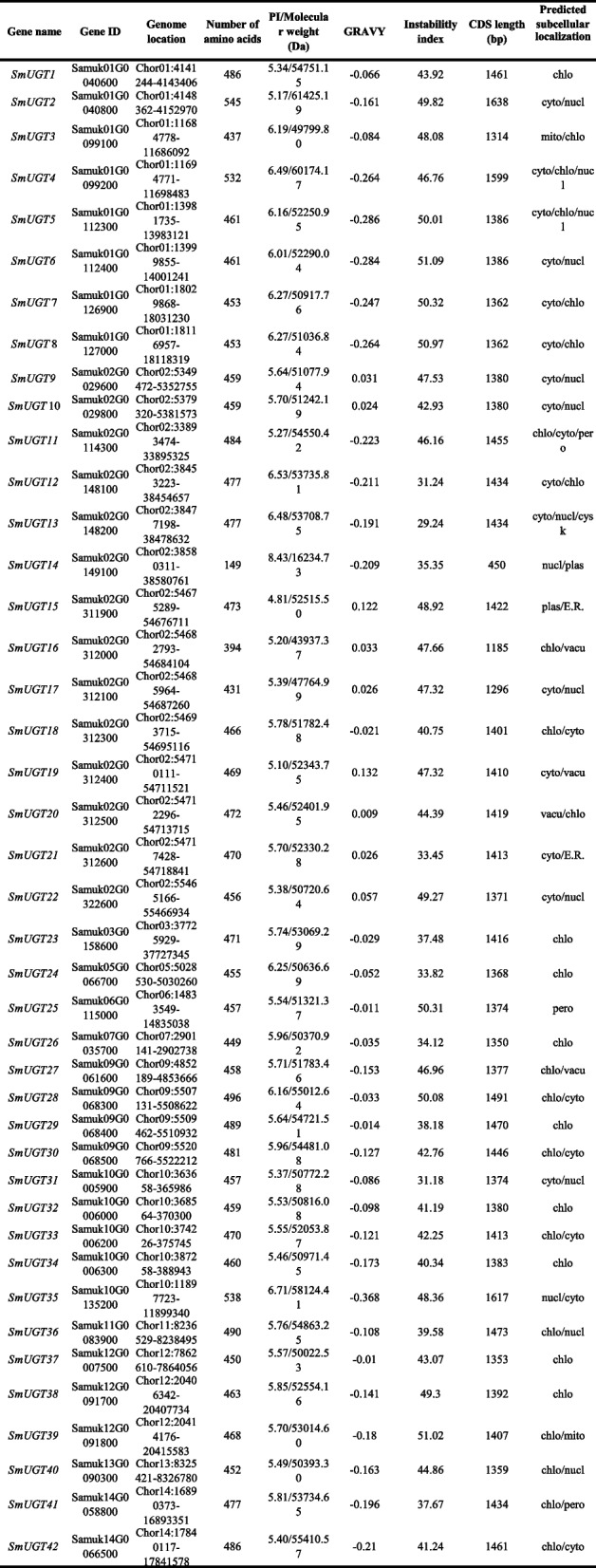
The physicochemical properties of the * SmUGT* genes

### Conserved domains of SmUGT proteins

**Fig. 2:**
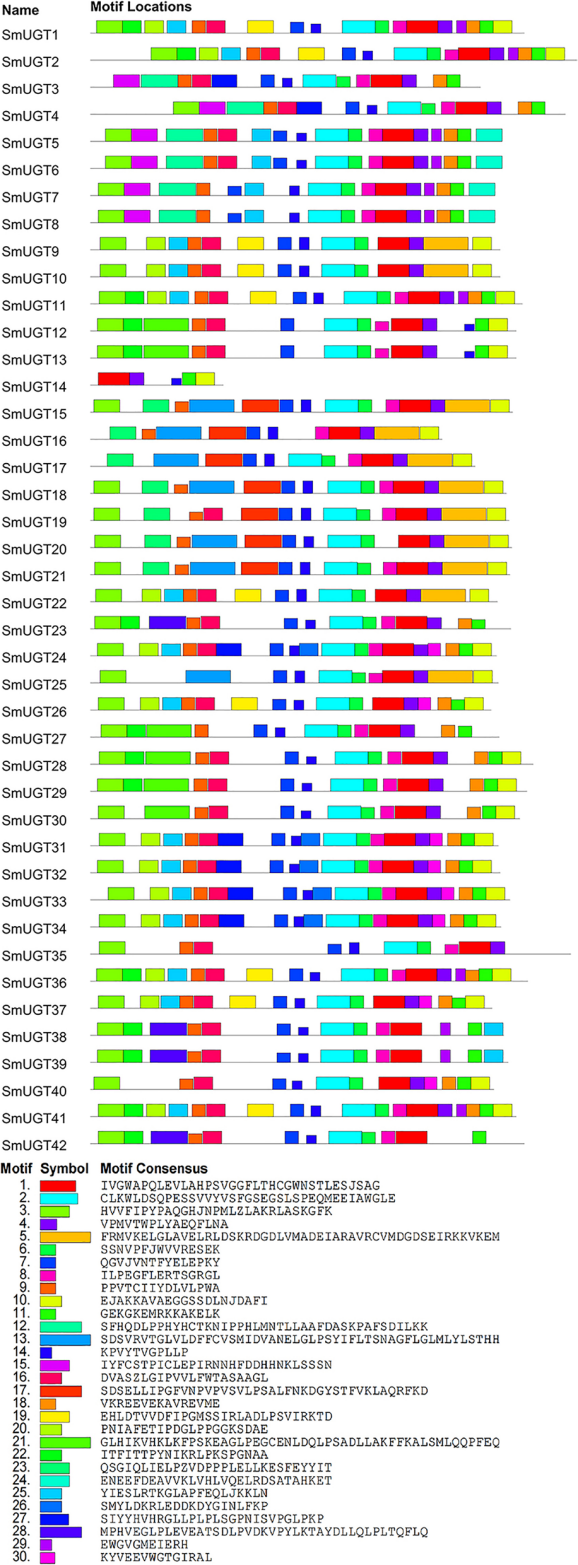
Conserved motifs of SmUGT proteins. The SmUGT sequences were visually analyzed; the top 30 motifs were selected and designated as motif 1 to motif 30

A sequence comparison performed in MEGA was visually analyzed in MEME v5.5.5, and the top 30 motifs were selected. The 42 SmUGTs harbored 5 to 18 motifs; all contained motif 1 and motif 4 (there was no complete motif 4 in SmUGT38, SmUGT39, and SUGT42 due to insertion of two amino acids in the sequence), the conserved PSPG-box region; all except SmUGT3, SmUGT14, SmUGT16, and SmUGT17 had motif 2 (Fig. 2). The PSPG-box is a highly conserved sequence of 44 amino acids with highly conserved positions 1 (W), 4 (Q), 7 (I/V), 8 (L), 12, 18 (S/A) (S/T), 19 (H) and 21 (G), 23 (N/S/G), 24 (S), 27 (E), 39 (P), 43 (E/D), and 44 (Q) [33]. SmUGT15-SmUGT21 and SmUGT25 had identical motif sequences, as did SmUGT31, SmUGT32, SmUGT33, and SmUGT34. Those motif sequences were almost identical in several proteins.

### Analysis of *cis*-acting elements of *SmUGT* genes

**Fig. 3:**
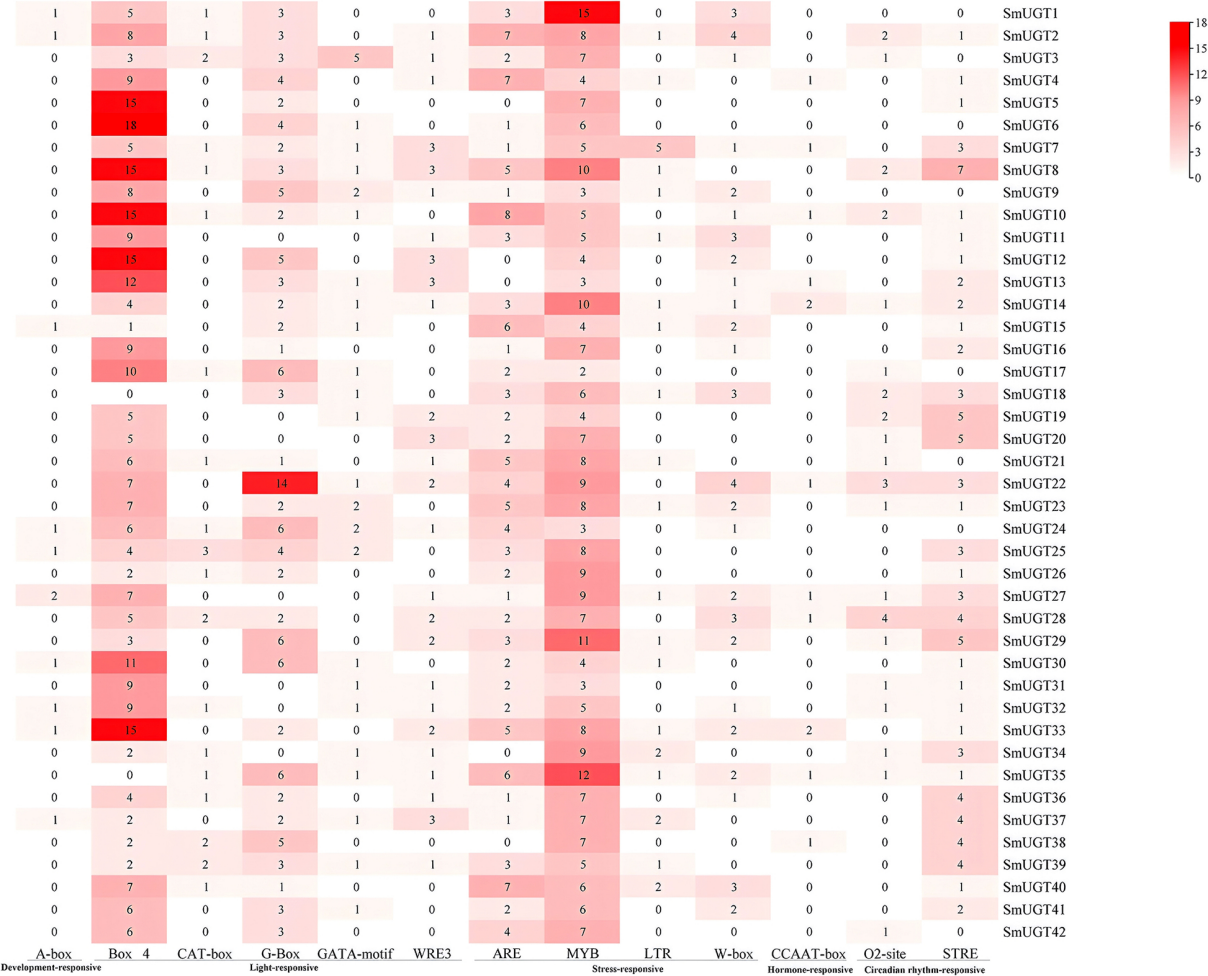
*cis-*Acting elements in a 3000 bp region upstream of *SmUGT* genes. Numbers represent the numbers of *cis*-acting elements; colors from white to red represent numbers from less to more

The* cis-*acting elements of *SmUGT*s with high frequencies (Figure S2) were screened and plotted (Fig. 3). The predicted functions of these elements were related to those identified by gene mining. They were categorized as elements responding to hormones, such as auxin, abscisic acid and gibberellin; stress response elements (e.g., light, drought, and oxygen); and elements regulating plant growth and development [34]. Among the selected elements, the developmental component was the A-box. Box 4, CAT-box, G-Box, GATA-motif, and WRE3 were related to plant photoperiod sensing; ARE, MYB, LTR, and W-box were associated with environmental stress regulation; O2-site and STRE were related to circadian rhythm regulation; and CCAAT-box was associated with hormone regulation. Among the 42 *SmUGT* promoters, 293 contained Box 4 and 280 had MYB. The numbers and positions of *cis-*acting elements differed among the *SmUGT*s (Figure S2).

### Phylogenetic analysis of SmUGT proteins

**Fig. 4:**
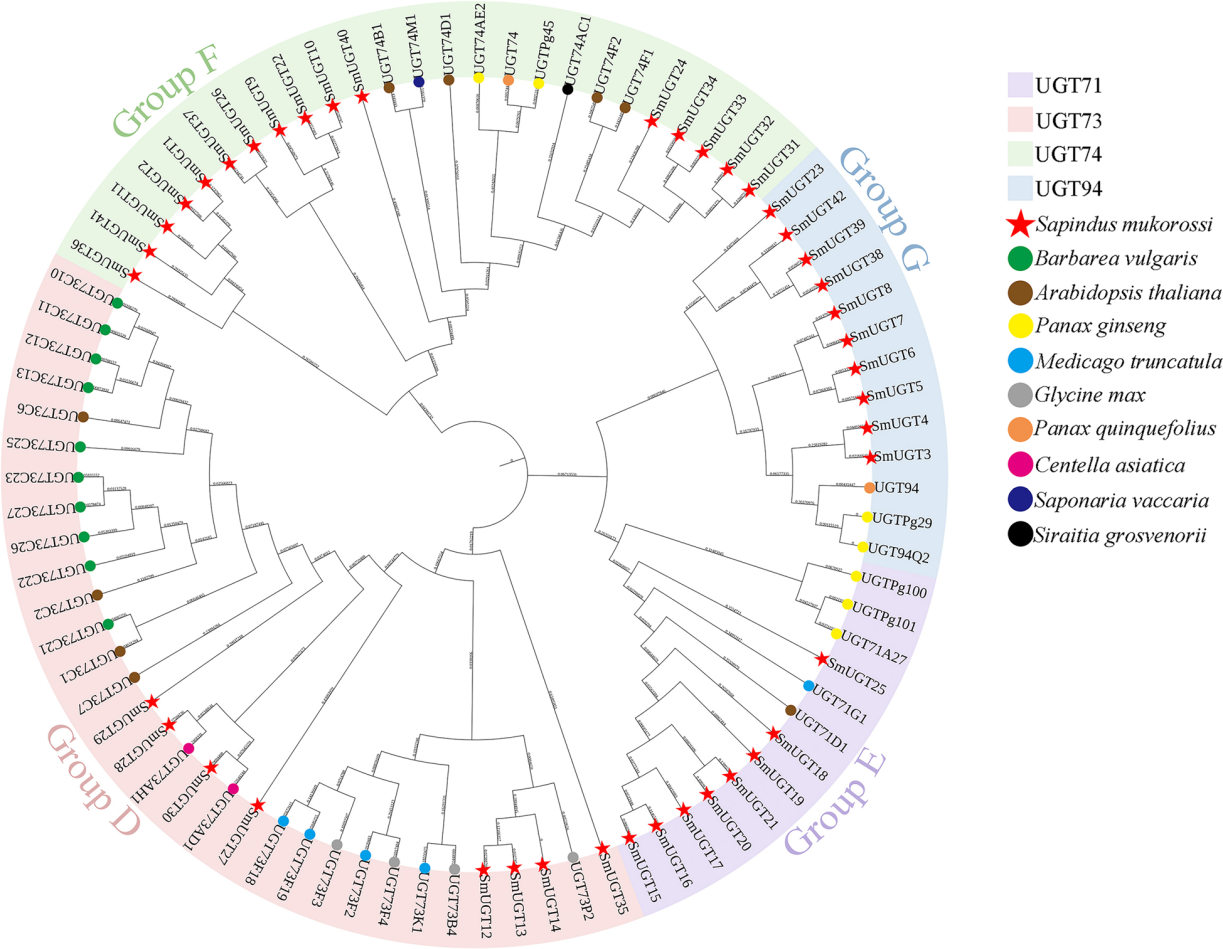
Phylogenetic analysis of SmUGTs. *Sapindus mukorossi* (42, red stars), *Barbarea vulgaris* (10, green circles), *Arabidopsis thaliana* (9, brown circles), *Panax ginseng* (7, yellow circles), *Medicago truncatula* (5, cyan circles), *Glycine max* (4, gray circles), *Panax quinquefolius* (2, orange circles), *Centella asiatica* (2, purple circles), *Saponaria vaccaria* (1, blue circle), and *Siraitia grosvenorii* (1, black circle)

We screened the UGT sequences of *Barbarea vulgaris*, *Arabidopsis thaliana*, *Panax ginseng*, *Medicago truncatula*, *Glycine max*, *Panax quinquefolius*, *Centella asiatica*, *Saponaria vaccaria*, and *Siraitia grosvenorii* using NCBI. A phylogenetic tree was constructed by combining their protein sequences with those of SmUGTs (Fig. 4).

The 42 SmUGTs belonged to the UGT71, UGT73, UGT74, and UGT94 subfamilies, classified into groups D, E, F, and G [33]. There were 8 members of UGT71, 8 of UGT73, 16 of UGT74, and 10 of UGT94. The 10 UGTs associated with triterpenoid saponin synthesis in *Barbarea vulgaris* were members of UGT73, whereas those in ginseng were members of UGT71, UGT74, and UGT94. In *Medicago truncatula*, four SmUGTs were members of UGT73 and one of UGT71. The four SmUGTs in soybean were members of UGT73.

**Fig. 5:**
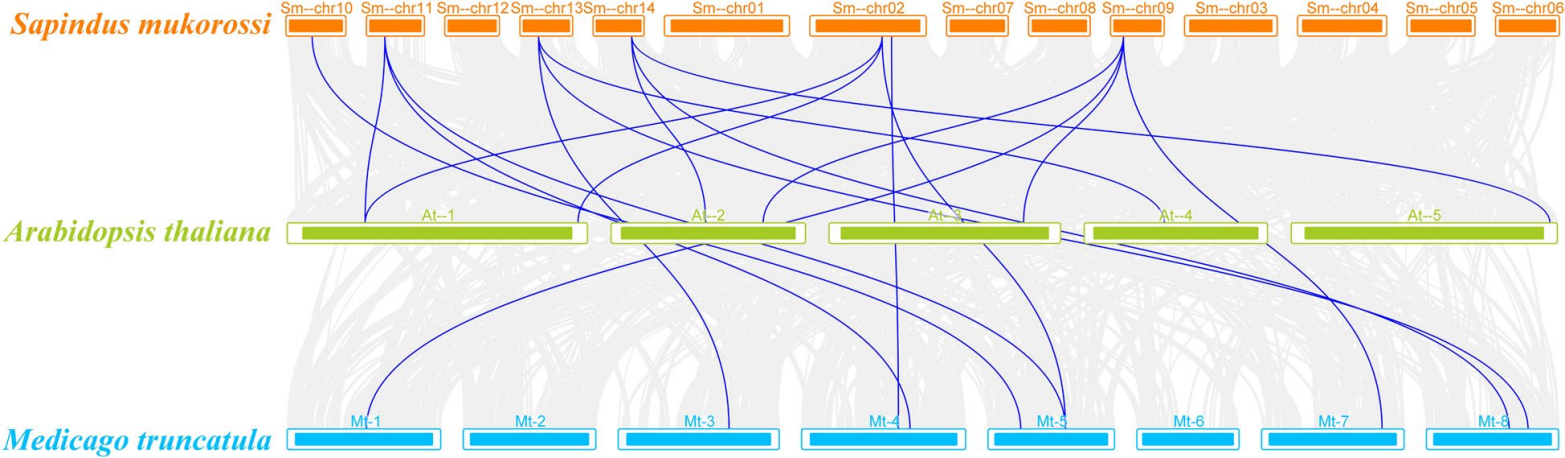
Synteny analysis of the *SmUGT* genes of soapberry and two other plant species. Gray lines in the background and red lines represent the collinear blocks and syntenic *UGT* gene pairs in the genomes of soapberry and other plant species, respectively

Collinearity analysis (Fig. 5) showed that 8 *SmUGT*s had collinear relationships with *UGT*s in *Arabidopsis thaliana* and 10 did with *UGT*s in *Medicago truncatula*; these were mainly distributed among chr02, chr09, chr10, chr11, chr13, and chr14. Therefore, soapberry is more closely related to *Medicago truncatula* than to *Arabidopsis thaliana*.

### Structural features of SmUGT proteins

The secondary structures of the SmUGTs consisted of an α-helix, extension strand, β-turn, and random coil (Table 2). SmUGTs harbored a large proportion of α-helices (41.70% average), followed by random coils (36.78%) and extended strands and β-turns (15.18% and 6.34%, respectively).

**Table 2.**
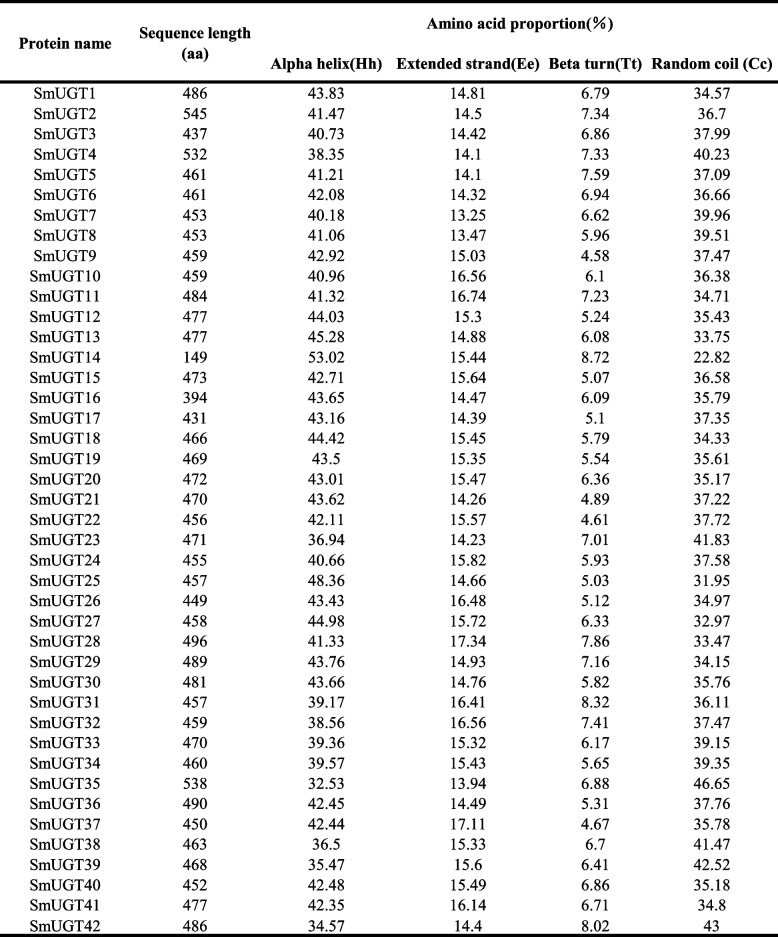
The secondary structure of the *SmUGT* proteins

**Fig. 6:**
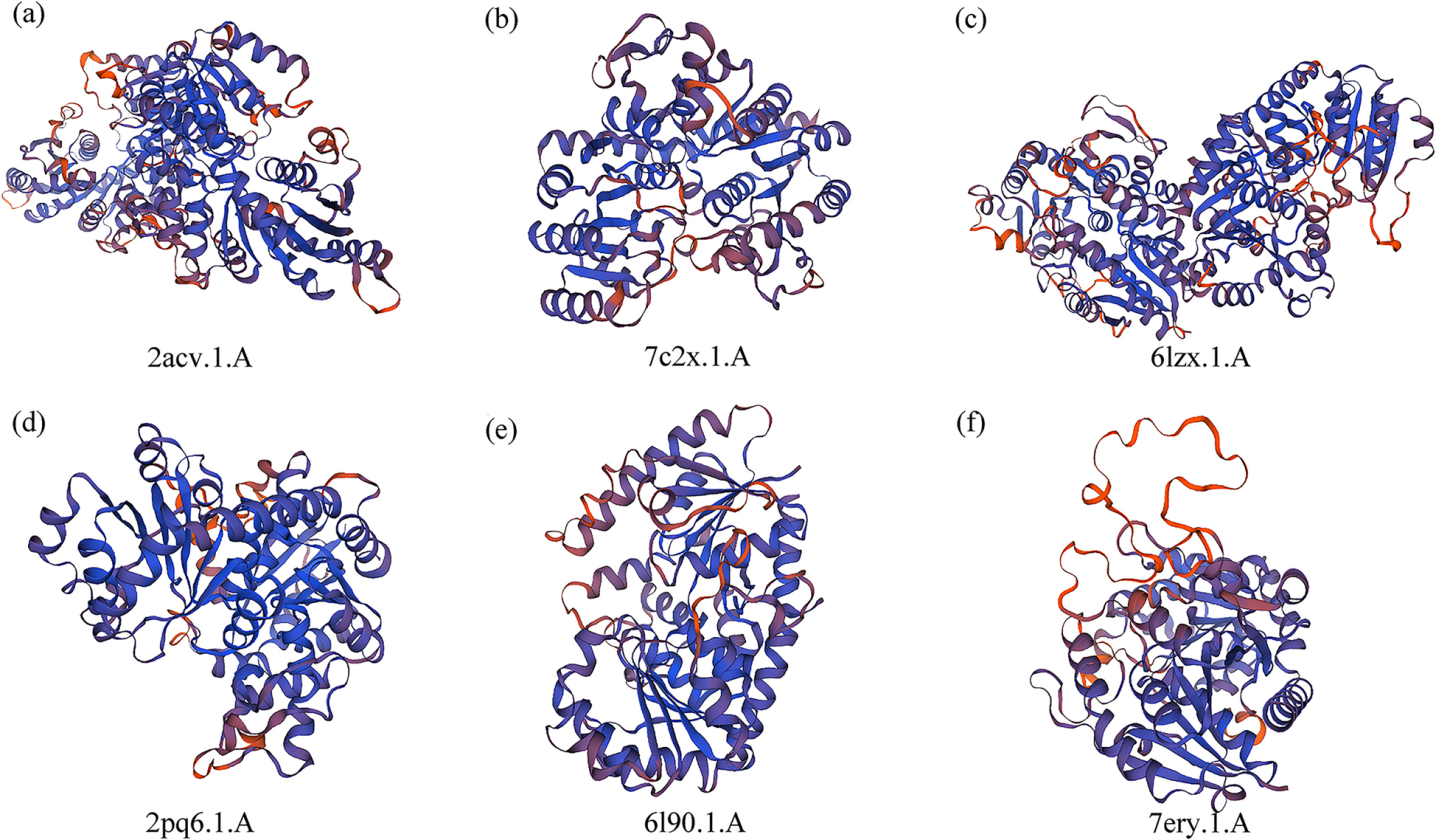
Three-dimensional model templates of several SmUGT proteins. **a** Prediction model templates for SmUGT15-SmUGT21 and SmUGT25 of UGT71 are both 2acv.1.A. **b** Prediction model templates for SmUGT12-SmUGT14 and SmUGT27 of UGT73 are 7c2x.1.A. **c** Prediction model templates for SmUGT28-SmUGT30 of UGT73 are all 6lzx.1.A. **d** Prediction model templates for SmUGT1, SmUGT2 and SmUGT11 of UGT74 are all 2pq6.1.A. **e** Prediction model templates for SmUGT24, SmUGT31-SmUGT34 are all 6l90.1.A. **f** Prediction model templates for SmUGT23, SmUGT39 and SmUGT42 of UGT74 are all 7ery.1.A

Images of the predicted 3D structural models were colored according to the N → C terminus. Several SmUGTs with the same predicted proteins were in the same subfamily (Fig. 6 and Table S1).

### *SmUGT* gene expression in tissues and at different developmental stages

**Fig. 7:**
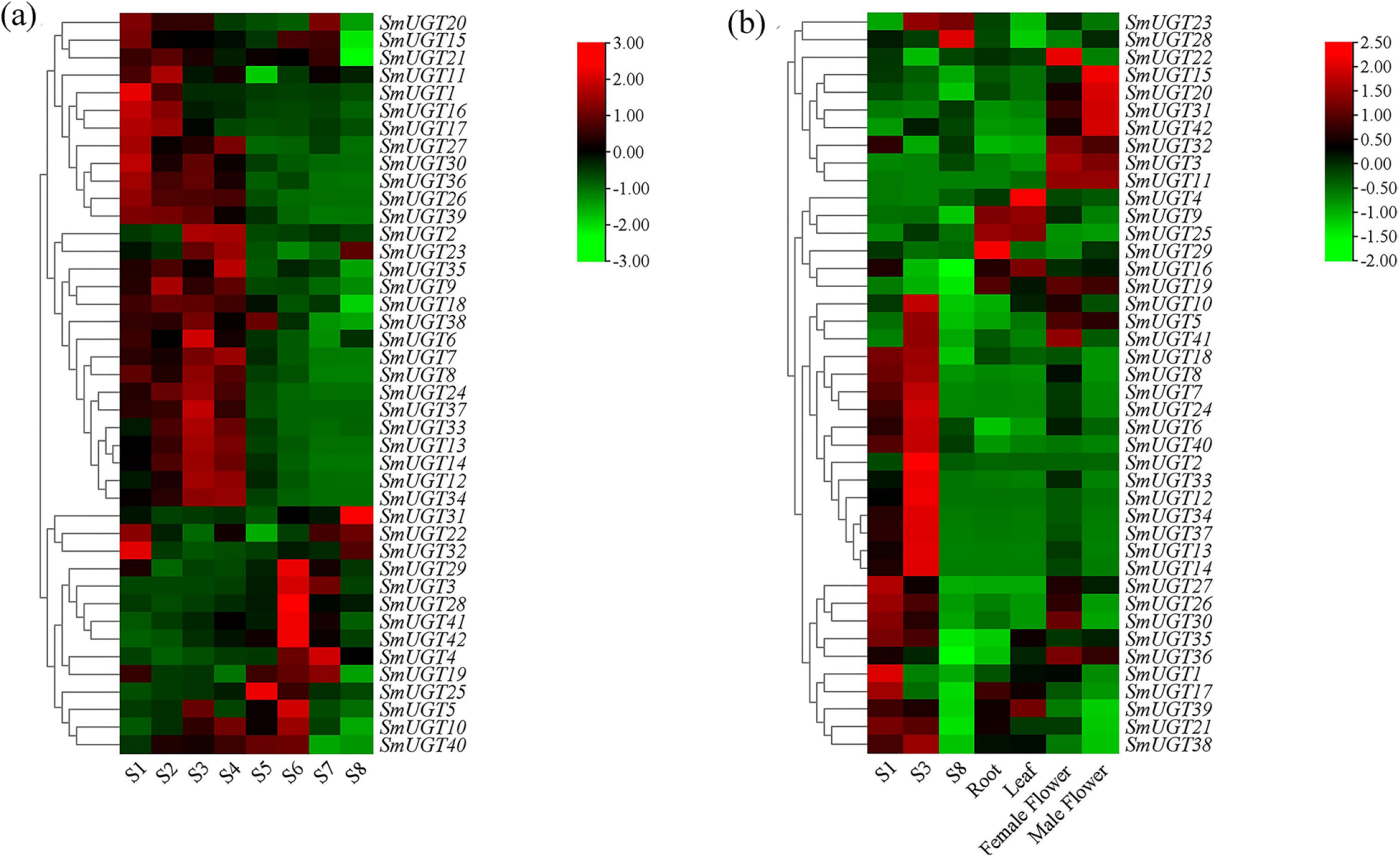
Expression levels of *SmUGT* genes during the indicated periods (**a**) and in the indicated tissues (**b**). The heat map showed red, black and green representing high, medium and low expression, respectively, and clustering related expression levels

The expression patterns of *SmUGT*s were explored based on transcriptome data obtained from fruit at eight developmental stages and from roots, leaves, and flowers; the results are shown as a heat map in Fig. 7.

According to the expression patterns during different stages of fruit development (Fig. 7a), *SmUGT*s were divided into two categories: those with expression levels higher from S1 to S4 than from S5 to S8 and those whose levels were higher from S5 to S8 than from S1 to S4. Most *SmUGTs* (93%) had low expression levels in S8, but those of *SmUGT22*, *SmUGT31*, and *SmUGT32* were significantly higher in S8. Few genes with high expression in S5 were found. Most genes were expressed at high levels in the reproductive organs, especially in female flowers and fruits early in development (Fig. 7b).

In the phylogenetic tree, the members of UGT71 had high expression levels from S1 to S4 and were downregulated from S5 to S8 (except *SmUGT19* and *SmUGT25*, which showed the opposite trend). Other than *SmUGT28* and *SmUGT29*, the members of UGT73 showed downregulation from S5 to S8. Three members of UGT74 were upregulated from S5 to S8. Four members of UGT94 were upregulated from S5 to S8. The expression levels of the 42 *SmUGT*s were high in female flowers at the S1 and S3 stages of fruit development but low in mature fruit (S8).

**Fig. 8:**
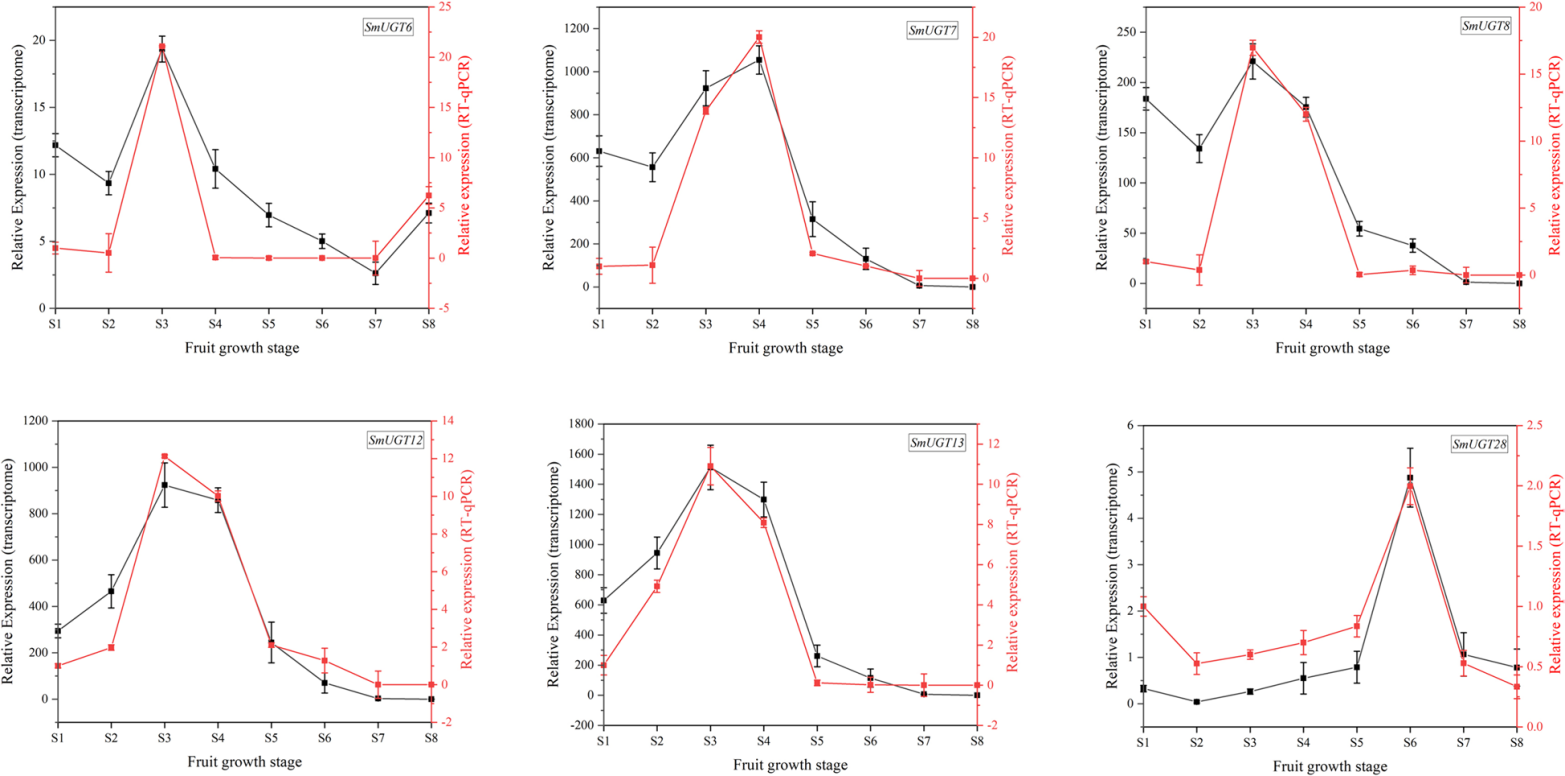
Validation of differential expression results obtained by RNA-seq. Relative expression levels determined by RT-qPCR and RNA-seq analyses of six selected genes at eight stages of fruit development

Validation by RT-qPCR showed that *SmUGT6*, *SmUGT7*, *SmUGT8*, *SmUGT12*, and *SmUGT13* were highly expressed from S1 to S4, whereas *SmUGT28* was highly expressed from S5 to S8 (Fig. 8). The results of RT-qPCR were essentially consistent with those of the transcriptomic analysis (Figure S3).

**Fig. 9:**
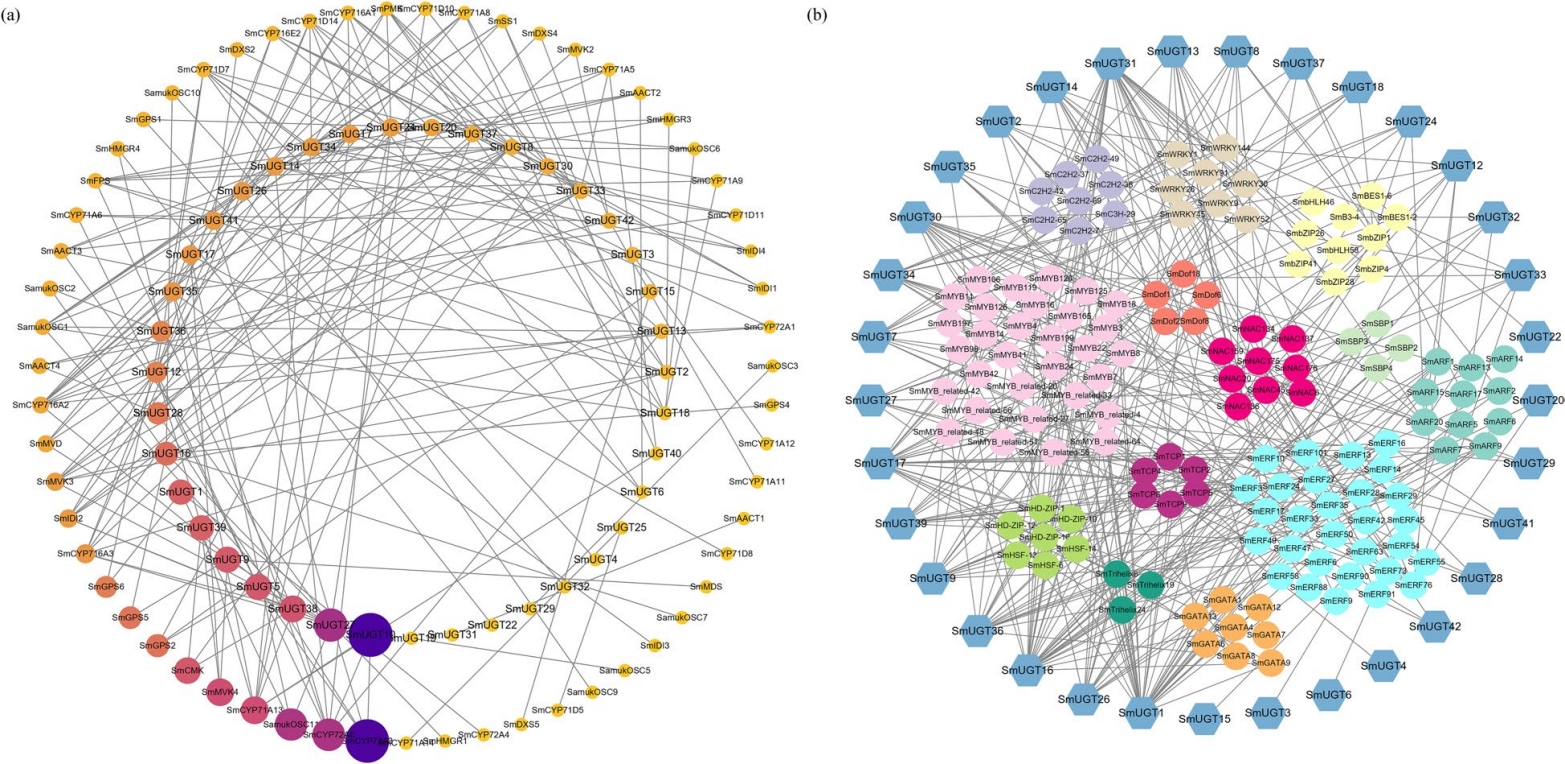
Coexpression network of *SmUGT*s and other genes (**a**) and transcription factors (b). **a** Colors range from orange to dark blue, and shapes vary from small to large representing the spectrum of gene expression strength. **b** Hexagons represent *SmUGT*s and circles represent transcription factors

As shown in Fig. 9, *SmUGT1*, *SmUGT5*, *SmUGT9*, *SmUGT10*, *SmUGT27*, *SmUGT38*, and *SmUGT39* were strongly correlated with other genes in the triterpenoid saponin synthesis pathway. *SmUGT1* and *SmUGT16* were strongly correlated with *SmERF*s, *SmGATA*s and *SmMYB*s, while *SmUGT31* was strongly correlated with *SmbZIP*s, *SmERF*s and *SmMYB*s.

### Relations between *SmUGT*s and saponins

**Fig. 10:**
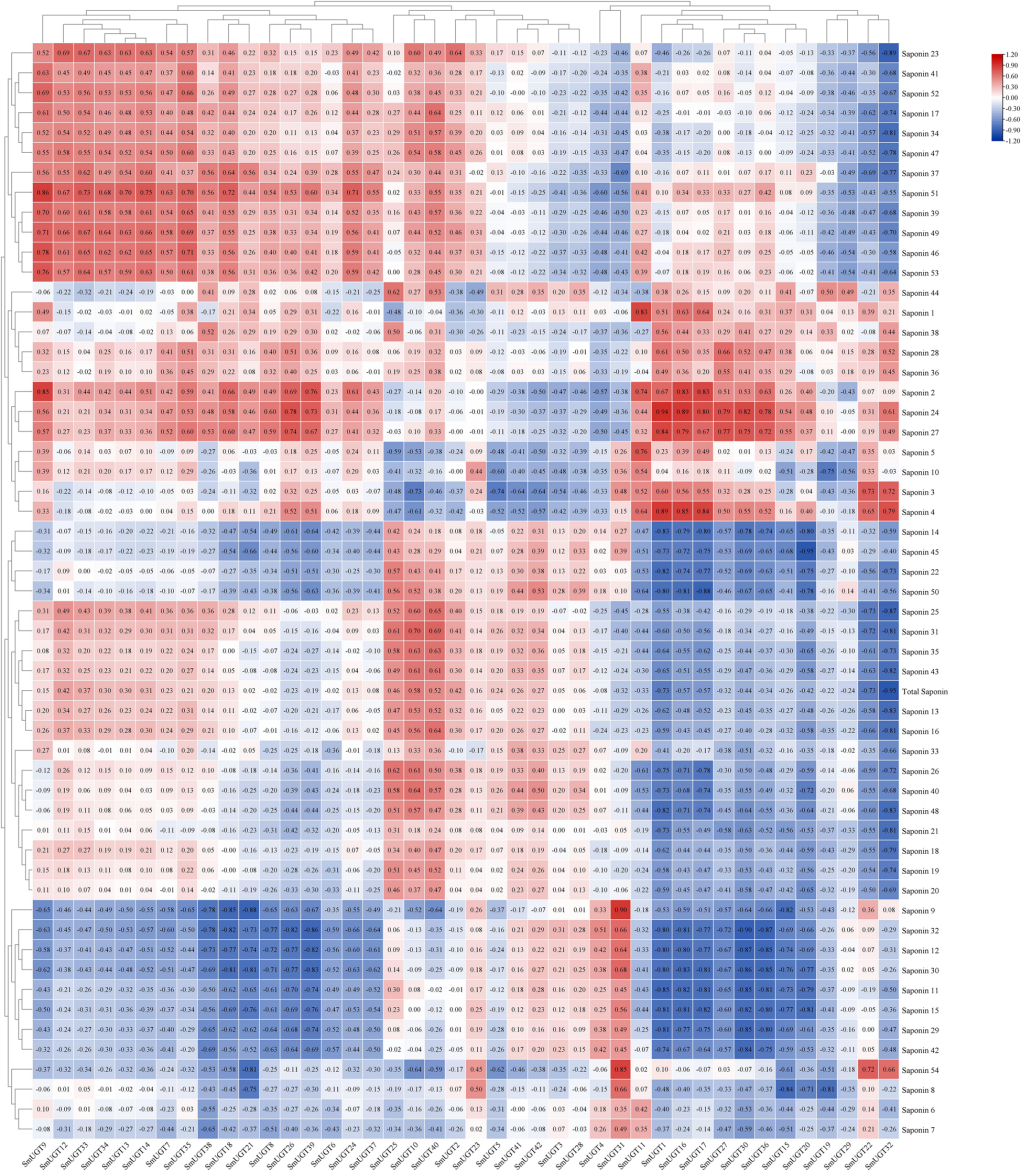
Correlation analysis between gene expression levels and saponin contents in soapberry. Red, white, and blue in the heat map represent positive, no, and negative correlation, respectively

We performed a correlation analysis of the contents of total saponins and monomeric saponins [32] (obtained previously, Table S3) with the changes in *SmUGT*s expression at the different stages of development (Fig. 10). Correlation clustering was also conducted.

There were strong positive correlations between Saponin 2 content and the expression levels of *SmUGT9*, *SmUGT16*, and *SmUGT17* during all stages. The content of Saponin 4 was positively correlated with the expression levels of *SmUGT1*, *SmUGT11*, *SmUGT16*, and *SmUGT17*; Saponin 9 and Saponin 54 with *SmUGT31*; Saponin 24 with *SmUGT1*, *SmUGT16*, *SmUGT17*, and *SmUGT30*; and Saponin 27 and Saponin 51 with *SmUGT1* and *SmUGT9*, respectively. In addition, the expression levels of *SmUGT32*, *SmUGT36*, and *SmUGT39* showed strong negative correlations with the contents of several saponins, particularly those of Saponin 30 and Saponin 32.

## Discussion

### Gene structural and conserved motifs of SmUGTs

Plant UGTs belong to the GT1 family and function in the synthesis of triterpenoid saponins. The members of this family have the conserved domain UDPGT (PF00201), whose C-terminal region contains a highly conserved sequence of 44 amino acids (PSPG-box). This sequence, together with the UDPGT domain, is a marker of UGTs. The sequence of the N-terminal region of UGTs is highly diverse, which explains their ability to recognize a variety of substrates [35]. The C-termini of the 42 SmUGTs harbored UDPGT domains (Figure S1) and PSPG-boxes (Fig. 2), indicating that the locations of the typical domains of this protein family were conserved among species. The specificity of the N-terminus of the SmUGTs protein was also noted. In addition, 97.6% of the SmUGTs were acidic and 78.6% were hydrophilic, which are similar to those of *Dimocarpus longan* [36], suggesting that SmUGTs are highly conserved proteins.

The position, loss, and gain of introns can be used to provide insight into the evolution of gene families within phylogenetic groups. Among the 42 genes, 45.2% had intron insertions (Fig. 1), a value lower than those of *Hippophae rhamnoides* (50.6%) [37], maize (60%) [20], and *Arabidopsis thaliana* (58%) [18], and similar to that of the Sapindaceae *Dimocarpus longan* (47.3%) [36]. Therefore, we speculate that intron insertion was gradually lost during the evolution of *SmUGT*s.

### Phylogenetic analysis of SmUGTs

UGTs are present in several gene families in plants, which are classified into 14 groups (A to N) in *Arabidopsis thaliana* [18]. The 42 SmUGTs in this study belonged to the subfamilies UGT71, UGT73, UGT74, and UGT94; they were divided into groups D, E, F and G. We evaluated the members of these groups in other species, among which group E is typically the largest [38]. In this study, 20% of SmUGTs belonged to group E (Fig. 4). Members of different groups exhibit different functional characteristics. Members of group D and group E can recognize a series of substrates, including terpenes [39]. Members of group F and group G are abundant in plants that produce triterpenoid saponins (e.g., *Panax ginseng* and *Panax quinquefolium* [23, 24]), and catalyze the glycosylation of substrates such as proginsenediol into ginsenosides. Therefore, it is speculated that these SmUGTs are related to glycosylation in the synthesis of triterpenoid saponins.

Phylogenetic analysis can provide insight into the evolutionary relationships among species and enable the identification of putative direct and collateral homologues. Homologous genes typically cluster into the same subgroup or subbranch and have similar functions [40]. The 10 UGTs associated with triterpenoid saponin synthesis in *Barbarea vulgaris* were members of UGT73 and catalyzed the 3-O-glucosylation of the sapogenins oleanolic acid and hederagenin [25]. Therefore, the genes of this subfamily (e.g., SmUGT12, SmUGT13) in soapberry may have similar functions. In *Medicago truncatula*, the function of glycosylation at C28/C3 of members of UGT71 and UGT73 subfamilies has been reported [26], so we performed a similar functional prediction for SmUGTs. The potential functions of SmUGTs will be further elucidated in future studies. As a woody plant, soapberry is characterized by a long growth cycle and the production of multiple types of saponin. The complex structure and low content of triterpenoid saponins make it difficult to obtain these molecules directly by chemical synthesis [5]. In the future, leveraging the function of SmUGTs, targeted and efficient production of soapberry triterpenoid saponins may be achieved by synthetic biology and bioreactor cultivation, leading to increased economic and practical value of SmUGTs.

The soapberry genes in each group were very similar and often clustered together. The *UGT* genes of herbs and woody plants may differ markedly. The proteins encoded by the clustered genes, e.g., SmUGT12, SmUGT13, and SmUGT14, showed consistent predicted 3D structures (Fig. 6 and Table S1), and shared a template.

### Expression patterns of *SmUGT*s at different stages of fruit development

The expression levels of genes at different developmental stages and in different organs can reflect their functions. The expression levels of most *SmUGT*s were high from S1 to S4 and low from S5 to S8 (Fig. 7). RT-qPCR validated the gene expression patterns during fruit development (Fig. 8). We speculate that most of the genes mediate glycosylation during the early stages of fruit development and promote the accumulation of saponins in fruit peel during the later stages. In other species, the expression of *UGT* genes changes as fruit development progresses. In *Hippophae rhamnoides*, most *UGT* genes are expressed at high levels in the early or middle stages of fruit development, and at lower levels in later stages [37]. In peach, 35% of *UGT* genes have the highest expression level in S1 (the first exponential growth phase, characterized by rapid cell division) [42]. This pattern is consistent with the expression patterns of most *SmUGT*s in our study. In *Gardenia jasminoides*, the expression levels of some *UGT* genes decrease with the development and maturation of fruits. However, the expressions of others increase with the continuous ripening of fruits [43].

We also analyzed the expression levels of *SmUGT*s in vegetative organs (roots and leaves) and reproductive organs (fruits, female and male flowers). Most of the genes were expressed at high levels in the reproductive organs, particularly in female flowers and in fruits in an early stage of development. Indeed, in *Medicago truncatula*, the expression of *UGT71G1* in flowers is higher than in roots, stems, and leaves [26]; many *UGT*s in maize are expressed at high levels in reproductive tissues, such as the anther and style [20]. In peach, the expression levels of 32% of *UGT*s are highest in flowers, and the abundances of 60 *UGT*s are highest during fruit development and postharvest maturation [42]. Therefore, *UGT*s are active in the reproductive organs of soapberry and the aforementioned plants, suggesting that in soapberry triterpenoid saponins are synthesized in flowers and fruits, and mainly accumulate in fruits.

An analysis of *cis-*acting elements suggested that light, hormones, and other factors modulate the expression of *UGT*s. In a previous study on *Epimedium pubescens*, the expression levels of > 12 *UGT* genes of group E and group D were significantly induced by intense light [44]. In a study on ginseng, MeJA promoted the expression of *UGT* and upregulated upstream terpenoid biosynthesis genes [41, 45]. *UGT* expression is induced by jasmonic acid in *Litsea cubeba* [46] and is downregulated by ABA stress in the roots of *Cajanus cajan* [47]. Therefore, exogenous light or hormones modulate the expression of *UGT* genes.

The expression levels of *SmUGT1*, *SmUGT9*, *SmUGT16*, and *SmUGT17* showed strong positive correlations with the contents of a variety of monomer saponins (Fig. 10), implicating these genes in saponin synthesis. In addition, several of those genes showed negative correlations with the contents of Saponin 30 and Saponin 32, possibly because of the synthesis of more complex triterpenoid saponins by glycosylation using these two monomeric saponins as receptor substrates.

## Conclusions

This study was the first to comprehensively analyze the *SmUGT*s related to triterpenoid saponin synthesis in *Sapindus mukorossi*, and 42 such genes were identified. These genes were unevenly localized on 12 chromosomes, their lengths ranged from 450 to 1638 bp, and their numbers of amino acids from 149 to 545. Most SmUGTs were acidic and unstable, hydrophilic proteins, and the secondary structures were mainly α-helices and random coils. The 42 SmUGTs were divided into four subfamilies, which showed sequence conservation and functional differences among them. Many types of *cis-*acting elements were involved in development, light response, and plant hormone response. The expression patterns of *SmUGT*s were significantly different among organs and developmental stages, and most were expressed during stages S1 to S4 of fruit development. In addition, we predicted the functions of *SmUGT*s based on their phylogenetic relationships and expression patterns. Our findings provide insight into the differences and genetic relationships among *SmUGT*s and lay a theoretical foundation for the functional study of key genes related to the synthesis of triterpenoid saponins in soapberry. In future studies, we will investigate the functions of SmUGTs to construct cellular factories for the targeted and efficient production of soapberry triterpenoid saponins.

## Materials and methods

### Plant materials

Three soapberry trees (6.5 m average height, 13.5 cm average diameter at breast height [DBH]) were cultivated at Soapberry National Forest Germplasm Banks in Jianning County, Fujian Province, China (26°49′ N latitude, 116°52′ E longitude, 300 m above sea level) [48]. We collected the roots, leaves, flowers and fruits samples between June and November 2018. Fruit samples were collected at eight growth stages: S1, early ovary development; 15 days after pollination (DAP); S2, 30% of largest fruit size, 45 DAP; S3, 70% of largest fruit size, 75 DAP; S4, 80% of largest fruit size, 90 DAP; S5, 90% of largest fruit size, 105 DAP; S6, beginning of maturity, 120 DAP; S7, great change in pericarp, 135 DAP; and S8, fully developed and mature, 150 DAP. Three biological replicates were taken at each stage, for a total of 24 samples. Fruits were randomly picked from the east, south, west, and north sides of the middle and upper parts of the crowns of trees at 10 a.m. on sunny days. After the fruit had been picked, the pericarp was immediately separated from the seed. A portion of each pericarp sample was transferred to liquid nitrogen and stored at − 80 °C for RNA extraction [32] The representative herbarium of *Sapindus mukurossi* in this study is deposited in Chinese Virtual Herbarium, the voucher number is PE 01391570.

### Identification and gene structural analysis

*SmUGT* genes were obtained from the *Sapindus mukorossi* reference genome annotation file (the complete genome of *Sapindus mukorossi* has been deposited in the National Genome Sciences Data Center (NGDC) database (https://ngdc.cncb.ac.cn). The whole genome GWH number WGS069104 can be accessed at PRJCA019364, and the whole genome was compared with the protein annotation information of *Glycine max* (https://www.soybase.org/sbt/) and *Medicago truncatula* (http://www.medicagogenome.org/) in NCBI (BLASTX; e ≤ 1 × 10^−5^). In all, 42 predicted *SmUGT*s were annotated. Gene structure maps of *SmUGT*s were generated using GSDS v2.0 (http://gsds.gao-lab.org/), and *SmUGT*s were mapped to soapberry chromosomes using TBtools v2.042. TBtools v2.042 was also used to perform collinearity analysis among soapberry, *Arabidopsis thaliana*, and *Medicago truncatula*. Pfam (http://pfam-legacy.xfam.org/) was used to predict conserved domains.

### Conserved motifs and phylogenetic analysis

ProParam (http://web.expasy.org/protparam) was used to analyze the physicochemical properties of SmUGT proteins and WoLF PSORT (https://wolfpsort.hgc.jp/) was used to predict their subcellular localizations. We used MEME v5.5.5 (http://meme-suite.org/) to predict motifs in candidate SmUGT amino acid sequences. We used MEGA7 software with the Muscle program to perform multiple sequence alignments and neighbor-joining (NJ) analyses, with the bootstrap value set to 1000 replicates.

### *cis*-acting element and protein structural analysis

PlantCARE was used to predict *cis*-acting elements in the 3000-bp upstream region (https://bioinformatics.psb.ugent.be/webtools/plantcare/html/). The secondary structures of SmUGTs were predicted using SoPMA (https://npsa-prabi.ibcp.fr/cgi-bin/npsa_automat.pl?page = npsa%20_sopma.html), and SWISS-MODEL (https://swissmodel.expasy.org/) was used for tertiary structure prediction.

### Expression patterns of *SmUGT* genes

We collected the vegetative organs (roots and leaves), reproductive organs (flowers and fruits), and peels from plants at different developmental stages and obtained high-quality FPKM transcriptome data [32]. The transcriptome data are available in the National Center for Biotechnology Information (NCBI) Sequence Read Archive (SRA) database. The accession number is PRJNA784159. Transcriptomic data were normalized using TBtools v2.042 to create a map of gene expression patterns. Plant materials were stored at − 80 °C. Samples were ground into powder with liquid nitrogen; total RNA was extracted using an RNA Rapid Extraction Kit, then reverse-transcribed into cDNA. RNA RT-qPCR was performed to verify the expression of six randomly selected *SmUGT*s at various developmental stages. The correlations between expression of *SmUGT*s and expression of other genes encoding triterpenoid saponin synthesis pathway/related transcription factors were analyzed. Results with Pearson correlation coefficient (*r*) > 0.6/0.9 were selected for mapping using Cytoscape software.

PCR was performed using a PerfectStart™ Green qPCR SuperMix Kit (Beijing Quantype Gold AQ602) on a LightCycler 480II Fluorescence Quantitative PCR Instrument (Roche, Basel, Switzerland). Primers were designed using Primer 3 (https://bioinfo.ut.ee/primer3-0.4.0/). *SmACT* was used as an internal reference [49], and expression levels were calculated using the 2^−∆∆Ct^ method. Correlations among expression profiles of six selected genes measured by RT-qPCR and RNA-seq were determined using origin v2019. Using the OmicShare cloud platform (https://www.omicshare.com/tools/Home/Soft/ica2), we analyzed the correlations of *SmUGT* expression levels with saponin contents in fruit at eight developmental stages.

### Supplementary Information

Supplementary Material 1.

Supplementary Material 2.

Supplementary Material 3.

Supplementary Material 4.

Supplementary Material 5.

Supplementary Material 6.

## Data Availability

The authors declare that the data supporting the findings of this study are available within the paper and its Supplementary Information files. Should any raw data files be needed in another format they are available from the corresponding author upon reasonable request.
