# Supplementary figures and images for: Identification and analysis of UGT genes associated with triterpenoid saponin in soapberry (Sapindus mukorossi Gaertn.)
Source: BMC Plant Biol. 2024 Jun 21;24:588. doi: 10.1186/s12870-024-05281-4 (PMC11191301; doi:10.1186/s12870-024-05281-4)

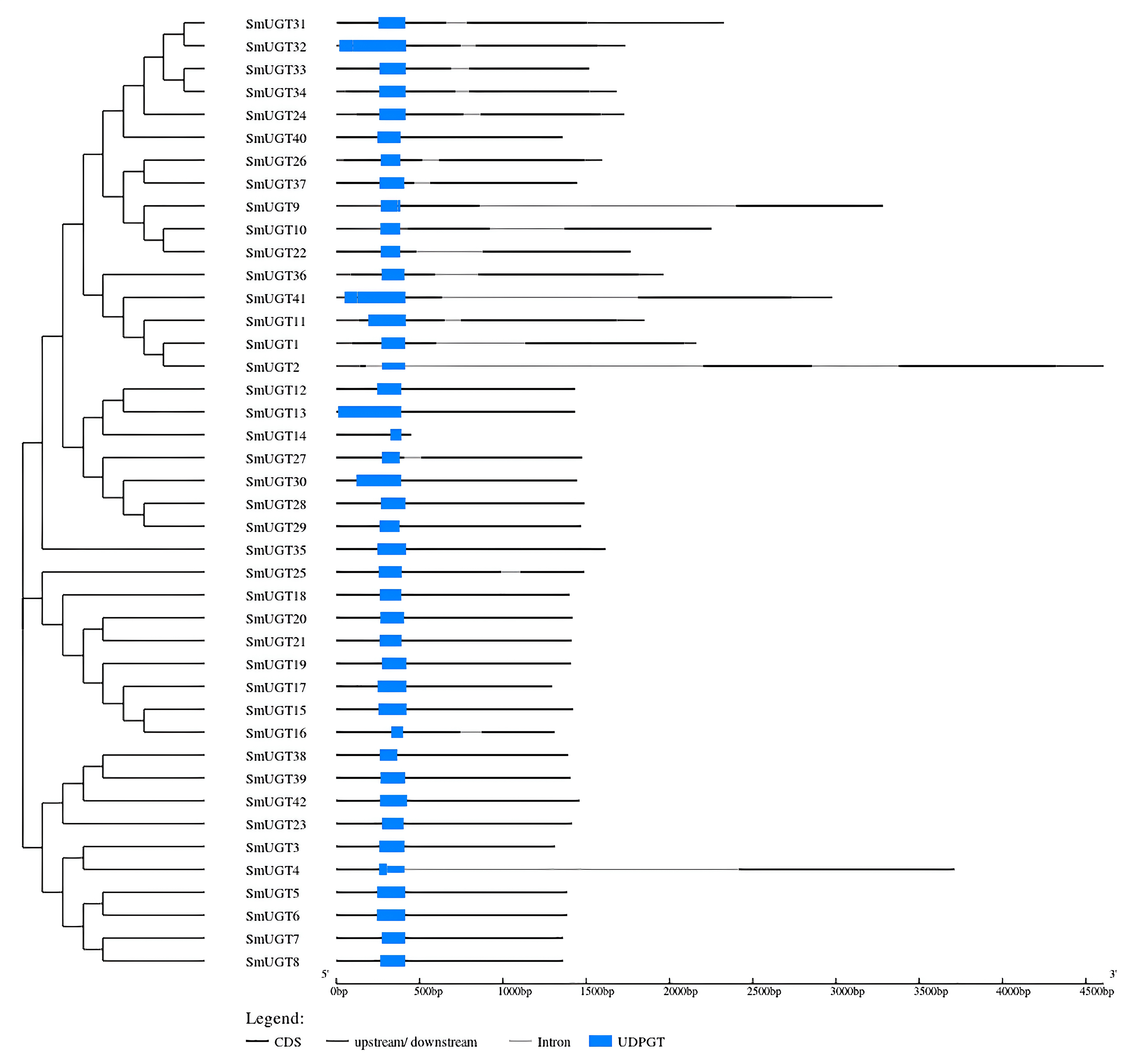

Supplement: Supplementary file 1 — Supplementary Material 1. [file 12870_2024_5281_MOESM1_ESM.jpeg]

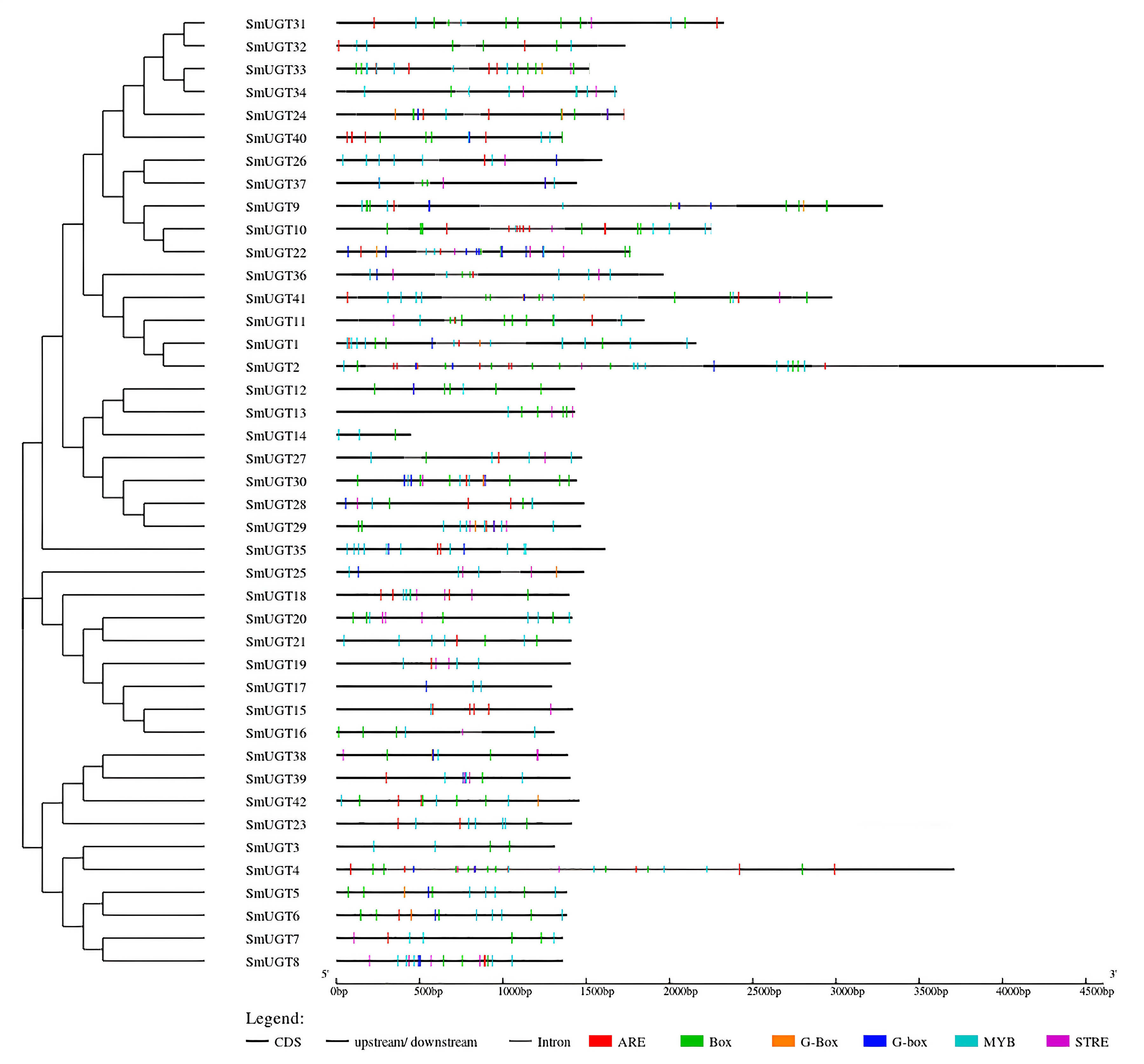

Supplement: Supplementary file 2 — Supplementary Material 2. [file 12870_2024_5281_MOESM2_ESM.jpeg]

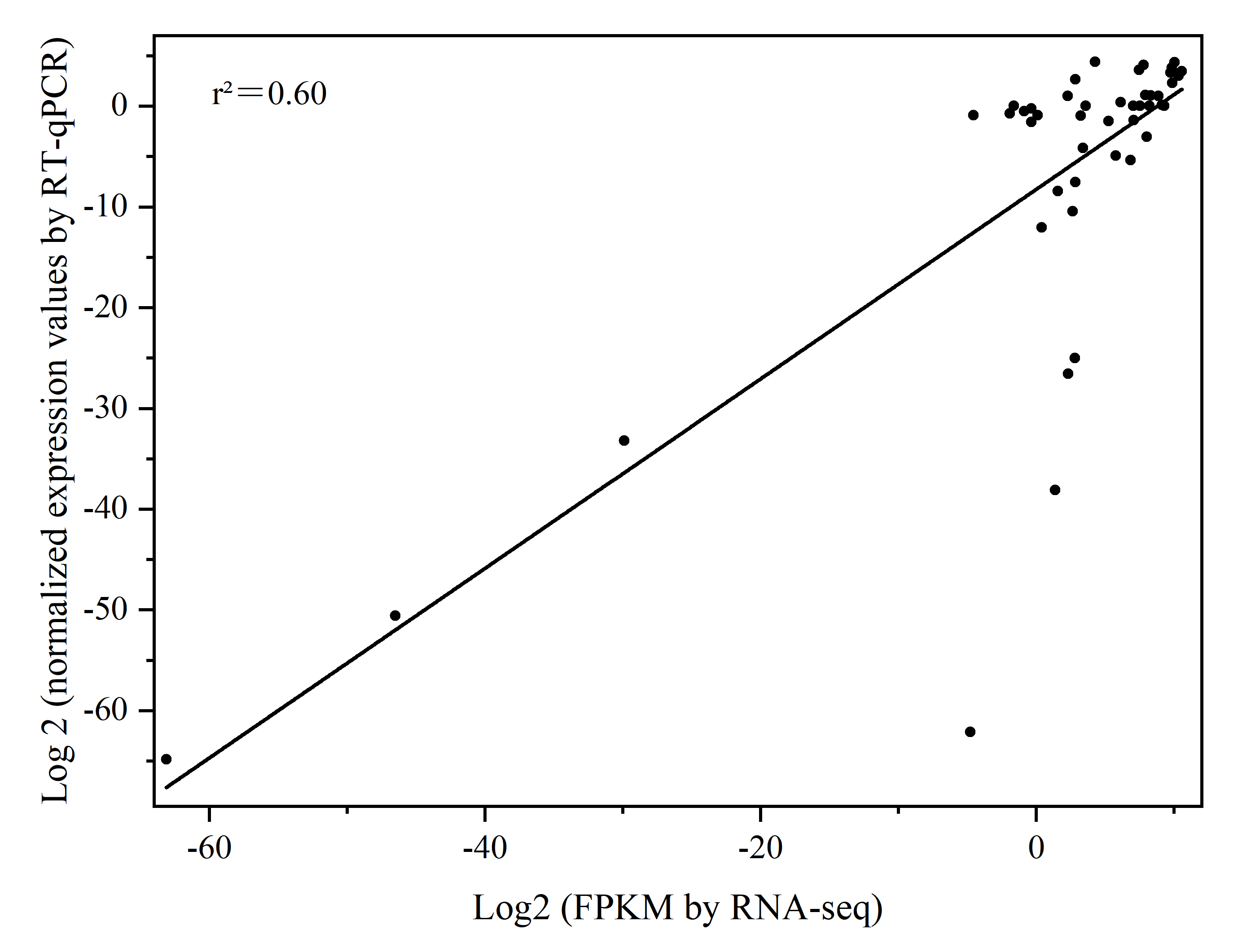

Supplement: Supplementary file 3 — Supplementary Material 3. [file 12870_2024_5281_MOESM3_ESM.jpeg]
