# Supplementary material for: Identification and analysis of UGT genes associated with triterpenoid saponin in soapberry (Sapindus mukorossi Gaertn.)
Source: BMC Plant Biol. 2024 Jun 21;24:588. doi: 10.1186/s12870-024-05281-4 (PMC11191301; doi:10.1186/s12870-024-05281-4)
Supplement: Supplementary file 4 — Supplementary Material 4. [file 12870_2024_5281_MOESM4_ESM.pdf]

Table S1 Three-dimensional structure annotation of SmUGT proteins

Protein name	Seq Identity (%)	GMQE	QMEAND	Template	Coverage	Range	Description
SmUGT1	54.53	0.83	0.82 ± 0.05	2pq6.1.A	0.98	6 ~ 478	UDP-glucuronosyl/UDP-glucosyltransferase
SmUGT2	52.97	0.73	0.79 ± 0.05	2pq6.1.A	0.87	67 ~ 537	UDP-glucuronosyl/UDP-glucosyltransferase
SmUGT3	31.01	0.64	0.65 ± 0.05	7erx.1.A	0.95	1 ~ 420	Glycosyltransferase
SmUGT4	27.42	0.53	0.64 ± 0.05	6lzx.1.B	0.84	87 ~ 531	Glycosyltransferase
SmUGT5	27.84	0.59	0.62 ± 0.05	7erx.1.A	0.93	17 ~ 450	Glycosyltransferase
SmUGT6	27.19	0.6	0.63 ± 0.05	2vg8.1.A	0.94	16 ~ 457	Hydroquinone Glucosyltransferase
SmUGT7	27.78	0.62	0.63 ± 0.05	6jtd.1.A	0.95	2 ~ 445	C-glycosyltransferase
SmUGT8	28.14	0.62	0.63 ± 0.05	6jtd.1.A	0.95	3 ~ 445	C-glycosyltransferase
SmUGT9	29.84	0.67	0.67 ± 0.05	7w0k.1.A	0.96	5 ~ 454	Glycosyltransferase
SmUGT10	30.45	0.67	0.66 ± 0.05	7w0k.1.A	0.96	4 ~ 454	Glycosyltransferase
SmUGT11	58.82	0.83	0.81 ± 0.05	2pq6.1.A	0.98	9 ~ 481	UDP-glucuronosyl/UDP-glucosyltransferase
SmUGT12	43.86	0.8	0.78 ± 0.05	7c2x.1.A	0.99	6 ~ 476	Glycosyltransferase
SmUGT13	42.8	0.79	0.77 ± 0.05	7c2x.1.A	0.99	6 ~ 476	Glycosyltransferase
SmUGT14	46.15	0.72	0.71 ± 0.07	7c2x.1.A	0.96	5 ~ 147	Glycosyltransferase
SmUGT15	56.5	0.79	0.78 ± 0.05	2acv.1.A	0.94	2 ~ 471	Triterpene UDP-glucosyl transferase UGT71G1
SmUGT16	55.77	0.77	0.78 ± 0.05	2acv.1.A	0.92	22 ~ 393	Triterpene UDP-glucosyl transferase UGT71G1
SmUGT17	52.35	0.71	0.72 ± 0.05	2acv.1.A	0.94	1 ~ 428	Triterpene UDP-glucosyl transferase UGT71G1
SmUGT18	48.65	0.78	0.76 ± 0.05	2acv.1.A	0.96	2 ~ 464	Triterpene UDP-glucosyl transferase UGT71G1
SmUGT19	54.48	0.79	0.77 ± 0.05	2acv.1.A	0.95	2 ~ 467	Triterpene UDP-glucosyl transferase UGT71G1
SmUGT20	52.47	0.78	0.77 ± 0.05	2acv.1.A	0.94	2 ~ 471	Triterpene UDP-glucosyl transferase UGT71G1
SmUGT21	51.69	0.77	0.76 ± 0.05	2acv.1.A	0.94	2 ~ 469	Triterpene UDP-glucosyl transferase UGT71G1
SmUGT22	29.52	0.67	0.67 ± 0.05	7w0k.1.A	0.96	2 ~ 451	Glycosyltransferase
SmUGT23	38.79	0.7	0.71 ± 0.05	7ery.1.A	0.95	3 ~ 465	Glycosyltransferase
SmUGT24	44.2	0.8	0.77 ± 0.05	6l90.1.A	0.98	7 ~ 453	Glycosyltransferase
SmUGT25	44.84	0.77	0.75 ± 0.05	2acv.1.A	0.98	4 ~ 454	Triterpene UDP-glucosyl transferase UGT71G1
SmUGT26	29.22	0.67	0.66 ± 0.05	7w0k.1.A	0.98	2 ~ 445	Glycosyltransferase
SmUGT27	37.73	0.72	0.71 ± 0.05	7c2x.1.A	0.96	10 ~ 447	Glycosyltransferase
SmUGT28	40.75	0.75	0.75 ± 0.05	6lzx.1.A	0.97	9 ~ 489	Glycosyltransferase
SmUGT29	44.23	0.77	0.77 ± 0.05	6lzx.1.A	0.98	7 ~ 484	Glycosyltransferase
SmUGT30	42.37	0.75	0.75 ± 0.05	6lzx.1.A	0.98	8 ~ 480	Glycosyltransferase
SmUGT31	47.79	0.78	0.77 ± 0.05	6l90.1.A	0.99	9 ~ 456	Glycosyltransferase
SmUGT32	44.69	0.77	0.76 ± 0.05	6l90.1.A	0.98	7 ~ 454	Glycosyltransferase
SmUGT33	46.33	0.78	0.76 ± 0.05	6l90.1.A	0.96	19 ~ 469	Glycosyltransferase
SmUGT34	47.22	0.81	0.77 ± 0.05	6l90.1.A	0.98	9 ~ 459	Glycosyltransferase
SmUGT35	26.16	0.53	0.58 ± 0.05	2vg8.1.A	0.84	9 ~ 536	Hydroquinone Glucosyltransferase
SmUGT36	51.68	0.8	0.80 ± 0.05	2pq6.1.A	0.97	6 ~ 477	UDP-glucuronosyl/UDP-glucosyltransferase
SmUGT37	28.83	0.67	0.65 ± 0.05	7w0k.1.A	0.97	2 ~ 446	Glycosyltransferase
SmUGT38	38.5	0.69	0.70 ± 0.05	7erx.1.A	0.95	8 ~ 460	Glycosyltransferase
SmUGT39	35.86	0.7	0.71 ± 0.05	7ery.1.A	0.96	6 ~ 461	Glycosyltransferase
SmUGT40	35.1	0.73	0.71 ± 0.05	5u6s.1.A	0.96	2 ~ 451	UDP-glycosyltransferase 74F2
SmUGT41	53.57	0.83	0.82 ± 0.05	2pq6.1.A	1	8 ~ 476	UDP-glucuronosyl/UDP-glucosyltransferase
SmUGT42	38.08	0.67	0.70 ± 0.05	7ery.1.A	0.92	7 ~ 466	Glycosyltransferase
