# Supplementary material for: Identification and analysis of UGT genes associated with triterpenoid saponin in soapberry (Sapindus mukorossi Gaertn.)
Source: BMC Plant Biol. 2024 Jun 21;24:588. doi: 10.1186/s12870-024-05281-4 (PMC11191301; doi:10.1186/s12870-024-05281-4)
Supplement: Supplementary file 5 — Supplementary Material 5. [file 12870_2024_5281_MOESM5_ESM.pdf]

Table S2 RT-qPCR gene-specific primer sequence list

Primer name	Sequence (5'to3')
FSmUGT6	GAGGAACTGGCAAATGTGGT
RSmUGT6	TGTTTCCTTGCAAGCAGTTG
FSmUGT7	TTGGGCTCCTCAAGCTAAAA
RSmUGT7	ATCTCCACCACCAACTTTGC
FSmUGT8	TTGGGCTCCTCAAGCTAAAA
RSmUGT8	ATCTCCACCACCAACTTTGC
FSmUGT12	AGCCCACTTTGTCCCCTACT
RSmUGT12	TGAGCCAGGTTTTCAATTCC
FSmUGT13	GATGCCACACGTAGACATGG
RSmUGT13	GAGGCCTTTCCCTATCTTGG
FSmUGT28	CTAATGTCACCAGGCCACCT
RSmUGT28	GTCACGTGAGGGAAGTTGGT
FSmACT	AGAAAGTTGGCCTCGCTGAA
RSmACT	CAGGAACCAGACCACCTGTC
