# Supplementary material for: Identification and analysis of UGT genes associated with triterpenoid saponin in soapberry (Sapindus mukorossi Gaertn.)
Source: BMC Plant Biol. 2024 Jun 21;24:588. doi: 10.1186/s12870-024-05281-4 (PMC11191301; doi:10.1186/s12870-024-05281-4)
Supplement: Supplementary file 6 — Supplementary Material 6. [file 12870_2024_5281_MOESM6_ESM.pdf]

Table S3 Triterpenoid saponin metabolites in soapberry

Meta ID	Identification	Molecular formula	Mass accuracy (×10^{-6})	Molecular weight (Da)	Class
Saponin 1	Mukurozi saponin Y1/Mukurozi saponin Y2	$C_{58}H_{94}O_{26}$	1.02853782	1206	Oleanane
Saponin 2	Mukurozi-saponin X or its isomers	$C_{53}H_{86}O_{22}$	0.83833712	1074	Oleanane
Saponin 3	saponin A or its isomers	$C_{46}H_{74}O_{16}$	0.77142172	882	Oleanane
Saponin 4	sapindoside C or its isomers	$C_{52}H_{84}O_{21}$	-2.5873323	1044	Oleanane
Saponin 5	saponin A or its isomers	$C_{46}H_{74}O_{16}$	4.85541906	882	Oleanane
Saponin 6	Mukurozi-saponin E1 or its isomers	$C_{48}H_{76}O_{17}$	2.58798087	924	Oleanane
Saponin 7	Mukurozi-saponin E1 or its isomers	$C_{48}H_{76}O_{17}$	2.19815949	924	Oleanane
Saponin 8	sapinmusaponin O/sapinmusaponin P	$C_{42}H_{72}O_{12}$	0.42295243	768	Dammarane
Saponin 9	saponin A or its isomers	$C_{46}H_{74}O_{16}$	0.97562159	882	Oleanane
Saponin 10	sapinmusaponin O/sapinmusaponin P	$C_{42}H_{72}O_{12}$	0.9425797	768	Dammarane
Saponin 11	saponin A or its isomers	$C_{46}H_{74}O_{16}$	2.64325384	882	Oleanane
Saponin 12	saponin A or its isomers	$C_{46}H_{74}O_{16}$	2.8474537	882	Oleanane
Saponin 13	hederagenin-3-O-Ara/Xyl-O-Rha-O-Rha	$C_{47}H_{76}O_{16}$	2.021206	896	Oleanane
Saponin 14	Mukurozi-saponin E1 or its isomers	$C_{48}H_{76}O_{17}$	-0.8554414	924	Oleanane
Saponin 15	hederagenin-3-O-Ara/Xyl-O-Rha-O-Rha	$C_{47}H_{76}O_{16}$	-4.7905932	896	Oleanane
Saponin 16	hederagenin-3-O-Ara/Xyl-O-Rha-O-Glc-OAc	$C_{49}H_{78}O_{18}$	0.14682574	954	Oleanane
Saponin 17	hederagenin-3-O-Ara/Xyl-O-Rha-O-Glc-OAc	$C_{49}H_{78}O_{18}$	3.03090282	954	Oleanane
Saponin 18	sapindoside A	$C_{41}H_{66}O_{12}$	2.2416527	750	Oleanane
Saponin 19	Mukurozi-saponin E1 or its isomers	$C_{48}H_{76}O_{17}$	2.6529511	924	Oleanane
Saponin 20	Mukurozi-saponin E1 or its isomers	$C_{48}H_{76}O_{17}$	-1.8408232	924	Oleanane
Saponin 21	Mukurozi-saponin E1 or its isomers	$C_{48}H_{76}O_{17}$	-4.223065	924	Oleanane
Saponin 22	Mukurozi-saponin E1 or its isomers	$C_{48}H_{76}O_{17}$	3.44342225	924	Oleanane
Saponin 23	Mukurozi-sapinin G or its isomers	$C_{50}H_{78}O_{18}$	-6.960049	966	Oleanane
Saponin 24	tetrahydrofuran tirucullane-O-Rha-O-Rha-O-Glc	$C_{48}H_{78}O_{16}$	1.4130371	910	Tirucullane
Saponin 25	Mukurozi-sapinin G or its isomers	$C_{50}H_{78}O_{18}$	0.24857318	966	Oleanane
Saponin 26	Mukurozi-sapinin G or its isomers	$C_{50}H_{78}O_{18}$	5.11646461	966	Oleanane
Saponin 27	tetrahydrofuran tirucullane-O-Rha-O-Rha-O-Glc	$C_{48}H_{78}O_{16}$	0.35068074	910	Tirucullane
Saponin 28	sapimukoside B or its isomers	$C_{42}H_{68}O_{12}$	7.61006399	763	Tirucullane
Saponin 29	Mukurozi-sapinin G or its isomers	$C_{50}H_{78}O_{18}$	4.73324763	966	Oleanane
Saponin 30	oleanolic acid 3-O-β-D-xylopyranosyl-(1→3) -α-L-rhamnopyranosyl-(1→2) -α-L-arabinopyranoside or its isomers	$C_{46}H_{74}O_{15}$	2.11439845	866	Oleanane
Saponin 31	Mukurozi-sapinin G or its isomers	$C_{50}H_{78}O_{18}$	0.69393346	966	Oleanane
Saponin 32	oleanolic acid 3-O-β-D-xylopyranosyl-(1→3) -α-L-rhamnopyranosyl-(1→2) -α-L-arabinopyranoside or its isomers	$C_{46}H_{74}O_{15}$	3.16582064	866	Oleanane
Saponin 33	Mukurozi-sapinin G or its isomers	$C_{50}H_{78}O_{18}$	2.23715862	966	Oleanane
Saponin 34	Rarasaponin IV or its isomers	$C_{52}H_{80}O_{19}$	2.18357732	1008	Oleanane
Saponin 35	oleanolic acid-3-O-Ac-Ara/Xyl-O-Rha-O-Ara/Xyl or tetrahydrofuran tirucullane-3-O-Ac-Ara/Xyl-O-Rha-O-Ara/Xyl	$C_{48}H_{76}O_{16}$	2.67767132	908	Oleanane/Tirucullane
Saponin 36	sapinmukoside B or its isomers	$C_{42}H_{68}O_{12}$	1.06862915	763	Tirucullane
Saponin 37	tetrahydrofuran tirucullane-O-Rha-O-Rha-O-Glc	$C_{48}H_{78}O_{16}$	0.90764427	910	Tirucullane
Saponin 38	sapinmukoside B or its isomers	$C_{42}H_{68}O_{12}$	0.92290699	763	Tirucullane
Saponin 39	Rarasaponin IV or its isomers	$C_{52}H_{80}O_{20}$	1.88581677	1008	Oleanane
Saponin 40	oleanolic acid-3-O-Ac-Ara/Xyl-O-Rha-O-Ara/Xyl or tetrahydrofuran tirucullane-3-O-Ac-Ara/Xyl-O-Rha-O-Ara/Xyl	$C_{48}H_{76}O_{16}$	1.79613343	908	Oleanane/Tirucullane
Saponin 41	Rarasaponin IV or its isomers	$C_{52}H_{80}O_{21}$	2.28283083	1008	Oleanane
Saponin 42	Mukurozi-sapinin G or its isomers	$C_{50}H_{78}O_{18}$	-16.882262	966	Oleanane
Saponin 43	oleanolic acid-3-O-Ac-Ara/Xyl-O-Rha-O-Ara/Xyl or tetrahydrofuran tirucullane-3-O-Ac-Ara/Xyl-O-Rha-O-Ara/Xyl	$C_{48}H_{76}O_{16}$	0.79338409	908	Oleanane/Tirucullane

Saponin 44	sapinmukoside B or its isomers	$C_{42}H_{68}O_{12}$	0.65574971	764	Tirucullane
Saponin 45	oleanolic acid-3-O-Ac-Ara/Xyl-O-Rha-O-Ara/Xyl or tetrahydrofuran tirucullane-3-O-Ac-Ara/Xyl-O-Rha-O-Ara/Xyl	$C_{48}H_{76}O_{16}$	1.99447946	908	Oleanane/Tirucullane
Saponin 46	Rarasaponin IV or its isomers	$C_{52}H_{80}O_{22}$	1.98507029	1008	Oleanane
Saponin 47	Rarasaponin IV or its isomers	$C_{52}H_{80}O_{23}$	-0.4267901	1008	Oleanane
Saponin 48	oleanolic acid-3-O-Ac-Ara/Xyl-O-Rha-O-Ara/Xyl or tetrahydrofuran tirucullane-3-O-Ac-Ara/Xyl-O-Rha-O-Ara/Xyl	$C_{48}H_{76}O_{16}$	3.34984395	908	Oleanane/Tirucullane
Saponin 49	Rarasaponin IV or its isomers	$C_{52}H_{80}O_{24}$	-2.004921	1008	Oleanane
Saponin 50	oleanolic acid-3-O-Ac-Ara/Xyl-O-Rha-O-Ara/Xyl or tetrahydrofuran tirucullane-3-O-Ac-Ara/Xyl-O-Rha-O-Ara/Xyl	$C_{48}H_{76}O_{16}$	2.07161402	908	Oleanane/Tirucullane
Saponin 51	Rarasaponin IV or its isomers	$C_{52}H_{80}O_{25}$	1.68730974	1008	Oleanane
Saponin 52	Rarasaponin IV or its isomers	$C_{52}H_{80}O_{26}$	13.5679554	1008	Oleanane
Saponin 53	Rarasaponin IV or its isomers	$C_{52}H_{80}O_{27}$	-3.7617082	1008	Oleanane
Saponin 54	tetrahydrofuran tirucullane-O-Rha-O-Rha	$C_{49}H_{80}O_{16}$	1.04721784	924	Tirucullane
